# The Metabolic Consequences of Hepatic AMP-Kinase Phosphorylation in Rainbow Trout

**DOI:** 10.1371/journal.pone.0020228

**Published:** 2011-05-20

**Authors:** Sergio Polakof, Stéphane Panserat, Paul M. Craig, David J. Martyres, Elisabeth Plagnes-Juan, Sharareh Savari, Stéphane Aris-Brosou, Thomas W. Moon

**Affiliations:** 1 INRA, UR1067 Nutrition Metabolism Aquaculture, Saint-Pée-sur-Nivelle, France; 2 Laboratorio de Fisioloxía Animal, Departamento de Bioloxía Funcional e Ciencias da Saúde, Facultade de Bioloxía, Universidade de Vigo, Vigo, Spain; 3 Department of Biology and Centre for Advanced Research in Environmental Genomics, University of Ottawa, Ottawa, Ontario, Canada; Oregon Health and Science University, United States of America

## Abstract

AMP-activated protein kinase (AMPK), a phylogenetically conserved serine/threonine protein kinase, is proposed to function as a “fuel gauge” to monitor cellular energy status in response to nutritional environmental variations. However, in fish, few studies have addressed the metabolic consequences related to the activation of this kinase. This study demonstrates that the rainbow trout (*Oncorhynchus mykiss*) possesses paralogs of the three known AMPK subunits that co-diversified, that the AMPK protein is present in the liver and in isolated hepatocytes, and it does change in response to physiological (fasting-re-feeding cycle) and pharmacological (AICAR and metformin administration and incubations) manipulations. Moreover, the phosphorylation of AMPK results in the phosphorylation of acetyl-CoA carboxylase, a main downstream target of AMPK in mammals. Other findings include changes in hepatic glycogen levels and several molecular actors involved in hepatic glucose and lipid metabolism, including mRNA transcript levels for glucokinase, glucose-6-phosphatase and fatty acid synthase both *in vivo* and *in vitro*. The fact that most results presented in this study are consistent with the recognized role of AMPK as a master regulator of energy homeostasis in living organisms supports the idea that these functions are conserved in this piscine model.

## Introduction

Intracellular ATP concentrations must be maintained within a narrow range to sustain appropriate metabolic function. Recent evidence indicates that the coordination of this task is achieved through the AMP-activated protein kinase (AMPK) [1]. This kinase is typically activated by an increase in the AMP:ATP ratio. In its active form, the AMPK regulates a large number of downstream targets that together lead to a reduction in anabolic pathways and a stimulation of catabolic pathways. The net result of AMPK activation is the stabilization of ATP levels and restoration of energy balance [2].

The AMPK of mammals exists as a heterotrimeric complex consisting of a catalytic α subunit and two regulatory subunits β and γ [1]. The conventional serine/threonine kinase activity of AMPK is supported by the α subunit, which is characterized by the presence of a threonine residue (Thr^172^) activation loop whose phosphorylation is required for full activation. Homologues of all three subunits exist in mammals, invertebrates, yeast, plants and the primitive protozoa, with a high degree of conservation suggesting that this signaling pathway evolved over one billion years ago to regulate metabolic homeostasis, although the exact evolutionary dynamics of AMPK are unclear [3].

The existence of AMPK is reported in several fish species including goldfish (*Carassius auratus*) [4], zebrafish (*Danio rerio*) [5], crucian carp (*Carassius carassius*) [6] and salmon (*Salmo salar*) (Markus Frederich and Jennifer Jost, unpublished observations). Additionally, numerous submissions to gene databases complete the list of species in which this kinase is reported including puffer fish (*Tetraodon nigroviridis*, *Takifugu rubripes*), stickleback (*Gasterosteus aculeatus*), and the Japanese ricefish (*Oryzias latipes*). Despite this apparent wide distribution in the piscine model, only a few studies have dealt with AMPK functions in fish or have established a phylogenetic relationship of AMPK subunits as found in fish and other vertebrates. Three different studies published nearly simultaneously addressed the role of AMPK in hypoxia-related functions all in hypoxic-insensitive cyprinids [4], [5], [6]. However, the only information regarding the metabolic consequences of the activation of this kinase in fish reported an increased metabolic rate in anoxic crucian carp treated with the AMPK inhibitor Compound C [6]; thus, further studies on AMPK function are warranted.

Mammalian AMPK is inactive unless phosphorylated by upstream kinases, with the critical phosphorylation site being Thr^172^ within the “activation loop” of the kinase domain within the α subunit [7]. Either 5′-AMP or the AICAR phosphorylated form (5-amino-4-imidazolecarboxamide ribotide [8]) activates AMPK by binding to the γ subunit [7]. This binding (i) promotes phosphorylation by the upstream kinase, (ii) allosterically activates the phosphorylated kinase, and (iii) inhibits dephosphorylation of Thr^172^ by protein phosphatases. There are at least two signaling pathways upstream of AMPK: one is triggered by an increase in the AMP:ATP ratio and dependent on LKB1, and the other is triggered by an increase in Ca^2+^ and is dependent on CaMKKβ [7]. Winder and Hardie [9] proposed that activators of AMPK may be effective treatments for type 2 diabetes. Later studies supported this idea, since AMPK was a potential target for biguanide antidiabetic drugs like metformin and phenformin, being activated in intact cells and/or *in vivo* through a LKB1-dependent mechanism [10], [11]. In fish, metformin is able to improve glucose homeostasis when administrated intraperitoneally (IP) [12], infused using osmotic pumps [13] or included in the food [14]. However, to date, the involvement of AMPK in this action remains to be elucidated.

The present study places the rainbow trout AMPK subunits into a phylogenetic context and shows that all three subunits diversified at approximately the same geological time. We employ two known activators of AMPK, AICAR and metformin (1,1-dimethylbiguanide hydrochloride) to explore the metabolic effects of the activation of AMPK in rainbow trout liver. Using mammalian antibodies, we identified in both trout liver and isolated hepatocytes the phosphorylation status of AMPK and indirectly assessed its activity by examining the phosphorylation of the AMPK downstream target acetyl-CoA carboxylase (ACC) at the serine residue (Ser^79^). We also conducted complementary experiments to establish whether physiological conditions modify trout liver AMPK, including feeding trout with metformin-supplemented diets or subjecting trout to a fasting-re-feeding cycle. Furthermore, we assessed hepatic glycogen levels and, at the molecular level, the activity of several representative genes of glucose and lipid metabolism in liver and isolated hepatocytes to explore the potential metabolic effects of AMPK activation.

## Materials and Methods

### Fish

Rainbow trout (*Oncorhynchus mykiss* Walbaum) were obtained from the INRA experimental fish farm facilities of Donzacq (Landes, France). Fish were maintained in tanks kept in open circuits supplied with 17°C well aerated water under a controlled photoperiod (LD 12:12) and fed with a commercial diet (T-3P classic, Trouw, France). Fish weighed 33±2 g for the AICAR experiments and 84±4 g for the metformin experiments. The experiments were conducted following the Guidelines of the National Legislation on Animal Care of the French Ministry of Research (Decree 2001-464 of May 29, 2001) and were approved by the Ethics Committee of INRA (according to INRA 2002-36 of April 14, 2002).

### Phylogenetic analysis

Sequences were downloaded from GenBank at the National Center for Biotechnology Information (www.ncbi.nlm.nih.gov). The human sequence of each paralog of each subunit was used as a BLASTn query to find all other sequences used in this study. A number of sequences from the chordates were retrieved, including a relatively large number of mammal sequences (i) to be able to use some of the fossil divergences as calibration priors (see below) and (ii) in order to check that accurate species trees were obtained for each paralog. Only sequences with an *E*-value <10^−500^ were used. The rainbow trout sequences were accessed by routine 5’RACE PCR and PCR experiments using human AMPK isoform primers. The resulting trout sequences were uploaded to GenBank (accession numbers: HQ403672-HQ403678).

The protein-coding parts of these sequences were extracted, and aligned with Muscle [15] for each subunit. The alignments were edited with Jalview [16]. The identity of the paralogs was checked with a preliminary phylogenetic analysis where each tree (for α, β and γ) was reconstructed under maximum likelihood using GTR + Γ_4_ (four discrete rate categories) and NNI + SPR in PhyML ver. 3.0 [17]. One thousand bootstrap replicates were generated to check the support for each internal bipartition. For all subsequent analyses, the best substitution model to be used for the analysis of each subunit was selected based on the Akaike Information Criterion as implemented in ModelTest [18].

The dated phylogenetic analysis relied on a relaxed molecular clock model as implemented in BEAST [19]. Fossil information was extracted from [20] with the selection of five divergences: *Amniota*/*Amphibia* (*Homo*/*Xenopus*: 0.330 billion years ago; BYA), *Mammalia*/*Sauropsida* (*Homo*/*Gallus*: 0.306 BYA), *Hominoidea*/*Cercopithecoidea* (*Homo*/*Macaca*: 0.020 BYA), *Theria*/*Prototheria* (*Homo*/*Ornithorhynchus*: 0.200 BYA) and *Actinopterygii*/*Sarcopterygii* (*Danio*/*Gallus*: 0.450 BYA). These fossil dates were used as minimum ages in the model, and mean 0.05 exponential prior distributions were used (note that times are in units of billion years). A vague lognormal prior (mean 0.5 and standard deviation 0.5) was placed on the root of each tree, with a minimum age of 0.450 BYA. The prior on rates was lognormal, and that on times was a pure-birth (Yule) process. The Markov chain Monte Carlo (MCMC) samplers were run in duplicates (to check convergence) for 100 million generations with a thinning of 2000 (see [21] for a brief introduction). Convergence was checked with Tracer (tree.bio.ed.ac.uk), which was also used to determine burn-in periods. After excluding the burn-in periods, each of the two replicate runs were combined and summarized with TreeAnnotator [19]. Note that no outgroup is required to root the tree when estimating divergence times (*e.g*., [22]).

### *In vivo* experimental protocols

For drug administration, 48 h-fasted fish were lightly anesthetized (0.05% (v/v) 2-phenoxyethanol), weighed, and intraperitoneally (ip) injected with 5 ml·kg^−1^ body mass saline solution alone (control, *n* = 6) or saline containing either AICAR (1.4 g·kg^−1^; Cayman) or metformin (120 mg·kg^−1^; Sigma). Sampling was initiated 2 h after the injection when six fish per group were randomly sampled and their liver immediately frozen in liquid nitrogen and kept at −80°C pending analyses.

The fasting-re-feeding trial was done with rainbow trout (fish mass 95±3 g) fasted for 10 days and then re-fed with a semi-purified diet (30% dextrin, 57% fish meal and 10% fish oil). Liver samples were taken before the re-feeding at 1 and 24 h after the meal. The nutritional study followed the experimental design described in Panserat et al. [14]. Briefly, two high carbohydrate and isocaloric diets were formulated and manufactured in the INRA facilities (30% gelatinized starch, 57% fish meal and 10% fish oil). One diet was supplemented with metformin (Merck, Paris, France) at a level of 0.25% while the other contained no metformin. Fish were hand-fed twice daily at 2% body weight for 10 days with the high-carbohydrate diet alone. After this period, fish were fed an additional 3 days with either the carbohydrate diet alone or the carbohydrate + metformin diet. At the end of the trial, six fish per group were sampled 2 h after the meal to follow the postprandial phase. All fish were killed by a sharp blow to the head and their liver removed and immediately frozen in liquid nitrogen and kept at −80°C until analyzed.

### *In vitro* experimental protocols

Isolated liver cells were prepared from four day-fasted rainbow trout as previously described by Mommsen and colleagues [23] with the modifications mentioned in Plagnes-Juan et al. [24]. Briefly, after anaesthesia, livers were perfused and digested *in situ*, excised and minced with a razor blade. After filtration and centrifugation (120 g, 2 min), the resulting cell pellets were resuspended three successive times in a modified Hanks’ medium (136.9 mM NaCl, 5.4 mM KCl, 0.81 mM MgSO_4_, 0.44 mM KH_2_PO_4_, 0.33 mM Na_2_HPO_4_, 5 mM NaHCO_3_ and 10 mM Hepes, pH = 7.63) and centrifuged (70 g, 2 min). Cells were finally suspended in the modified Hanks’ medium supplemented with 1.5 mM CaCl_2_, 1% defatted BSA, 4·10^−9^ M bovine insulin (Sigma), 20 mM glucose, MEM essential amino acids (1x; Invitrogen), MEM non-essential amino acids (1x; Invitrogen) and antibiotic antimycotic solution (1x; Sigma). Hepatocytes were plated in six well culture dishes at a density of 3·10^6^ cells per well and incubated at 18°C. The incubation medium was replaced every 24 h over the 72 h primary cell culture period. For protein analysis, 48 h-cultured hepatocytes were stimulated using either metformin or AICAR at 0.5 mM for 16 h, doses used previously for mammalian cell culture studies [25], [26]. At the end of the stimulation period, cells were collected for protein extraction.

### Western blot and qPCR analysis

Protein extraction (20 µg) and Western blotting were undertaken as in [13] using anti-phospho-AMPKα Thr^172^ and anti-AMPK antibodies (Cell Signaling Technology, France) that recognize both α-1 and α-2 isoforms of the enzyme. For ACC, Akt and β-tubulin blots, anti-phosphor-ACC Ser^79^ and anti-β-tubulin antibodies (Cell Signaling Technology, France) were used as noted previously. Frozen livers or hepatocytes were homogenized on ice with an Ultraturrax homogenizer in a buffer containing 150 mM NaCl, 10 mM Tris, 1 mM EGTA, 1 mM EDTA (pH = 7.4), 100 mM sodium fluoride, 4 mM sodium pyrophosphate, 2 mM sodium orthovanadate, 1% Triton X-100, 0.5% NP-40-IGEPAL and a protease inhibitor cocktail (Roche, Basel, Switzerland). Homogenates were centrifuged for 15 min at 12,000 g and the resulting supernatants stored at −80°C. Protein concentrations were determined using the Bio-Rad protein assay kit (BIO-RAD, Hercules, CA, USA). Protein lysates (20 µg of protein) were subjected to SDS-PAGE and Western blotting using the appropriate antibody. After washing, membranes were incubated with an IRDye infrared secondary antibody (LI-COR Inc. Biotechnology, Lincoln, NE, USA). Bands were visualized by Infrared Fluorescence using the Odyssey® Imaging System (LI-COR Inc. Biotechnology, Lincoln, NE, USA) and quantified by Odyssey infrared imaging system software (Application software, version 1.2).

Liver mRNA levels for GK (glucokinase), G6Pase (glucose 6-phosphatase), FAS (fatty acid synthase), PEPCK (phosphoenolpyruvate carboxykinase) and FBPase (fructose 1,6-bisphosphatase) were estimated by real-time quantitative RT-PCR (q-PCR) [13]. Primers were designed to overlap an intron where possible (Primer3 software) using known sequences found in trout nucleotide databases (GenBank and INRA-Sigenae) and AMPK partial sequences (see Table 1) as previously described [13]. Quantification of the target gene transcript level was done using *ef1α* gene expression as reference, which was found to be stably expressed in this study. Relative quantification of the target gene transcript with the *ef1α* reference gene transcript was made following the Pfaffl method [27].

**Table 1:** Sequences of the primer pairs used for real-time quantitative PCR determination of the transcript levels of several rainbow trout genes involved in glucose, lipid and energy metabolism.

Gene	5′-3′ forward primer	5′-3′ reverse primer
***SybrGreen***		
GK	TGAAGGATCAGAGGTGGGTGAT	GAAGGTGAAACCCAGAGGAAGC
G6Pase	CTCAGTGGCGACAGAAAGG	TACACAGCAGCATCCAGAGC
FBPase	GCTGGACCCTTCCATCGG	CGACATAACGCCCACCATAGG
PEPCK	GTTGGTGCTAAAGGGCACAC	CCCGTCTTCTGATAAGTCCAA
FAS	GAGACCTAGTGGAGGCTGTC	TCTTGTTGATGGTGAGCTGT
EF1 α	TCCTCTTGGTCGTTTCGCTG	ACCCGAGGGACATCCTGTG
***TaqMan***		
AMPK α 1	CACCATCAAAGAGATCCGAGAG	TCAAACTTCTCACACACCTCC
	Probe:/56-FAM/ATGAGCTCC/ZEN/CCAAGTACCTGTTTCC/3IABKFQ/	
AMPK α 2	GGGCTACCATTAAAGACATTAGGG	ACTCGGTGCTCTCAAACTTG
	Probe:/56-FAM/TCGGACTGC/ZEN/CTCCTCATCCAGA/3IABKFQ/	
β-actin	CAATGAGACTGAGAAGCTGGG	GACTGTACCCATCCCAAACG
	PROBE: FAMCCACACCCGACTACCACTTCAGC/3IABKFQ	

GenBank accession no. or sigenae accession no.: EF1α (elongation factor 1α), AF498320; GK (glucokinase), AF135403; PEPCK (phospho*enol*pyruvate carboxykinase), AF246149; G6Pase (glucose 6-phosphatase), tcay0019b.d.18_3.1.s.om.8.1-1693; FBPase (fructose 1,6-bisphosphatase), AF333188; FAS (fatty acid synthase), tcab0001c.e.06_5.1.s.om.8; AMPK α 1, HQ403672; AMPK α 2, HQ403673.

Due to the similarities of the AMPKα1 and AMPKα2 isoforms, probe-based qPCR was employed to detect differences in expression from liver samples. Briefly, first strand cDNA was synthesized using QuantiTect Reverse Transcription Kit (Qiagen). mRNA expression was quantified in duplicate on a Stratagene MX3000P real-time PCR machine using probe based (FAM labeled) PrimeTime Mini qPCR Assays (Integrated DNA Technologies). Each reaction contained 12.5 µl SYBR green mix, 1 µl primer/probe mix, 0.375 µl ROX reference dye (1∶500 dilution), 10.125 µl RNase/DNase-free H_2_O, and 1 µl of 5x diluted cDNA template. Cycling conditions were as follows: 10 min initial denaturation at 95°C, 40 cycles at 95°C for 30 s, and 60°C for 1 min. To account for differences in amplification efficiency between different cDNAs, standard curves were constructed for each target gene using serial dilutions of quantified liver and muscle cDNA. To account for differences in cDNA production and loading differences, all samples were normalized to the expression level of the housekeeping gene β-actin, which did not change over the course of the experimental treatments. Gene expression data were calculated using the 2^ΔΔ^- method of Pfaffl [27]. Both RNase/DNase-free H_2_O and non-reverse transcribed RNA were assayed on each plate to ensure the absence of contamination in the reagents and primers used. Primers were designed using Integrated DNA Technologies online assay design program and can be found in Table 1.

### Statistical analysis

Results are expressed as means ± s.e.m. (*n* = 6) and were analyzed by one-way ANOVA to compare groups receiving drug administration or the vehicle using Student-Newman-Keuls *a posteriori* multiple comparison test. The level of significance was set at *α*<0.05.

## Results

### Phylogeny of AMPK subunits

Rainbow trout AMPK genes were sequenced for the α subunit (α1: HQ403672, α2: HQ403673), β subunit (β1.1: HQ403674; β2: HQ403675) and γ subunit (γ1: HQ403676, γ2.1: HQ403677, γ2.2: HQ403678). The dot notation for the fish-specific paralogs is ours. No γ3 sequences were obtained. The actual identification of these sequences was performed through a phylogenetic analysis, conducted as described in the Materials and Methods. The BLASTn searches (conducted in November 2010) for AMPK subunits returned 52 α sequences, 52 β sequences and 76 γ sequences. The only γ3 sequence found in fish was that of *Gasterosteus aculeatus*; no other fish γ3 sequences were found, even with a more sensitive tBLASTx search. The preliminary phylogenetic analysis led us to eliminate isoforms due to transcript variants, duplicates and sequences of poor quality, resulting in three final data sets comprising 47 α sequences, 47 β sequences and 73 γ sequences.

**Figure 1:**
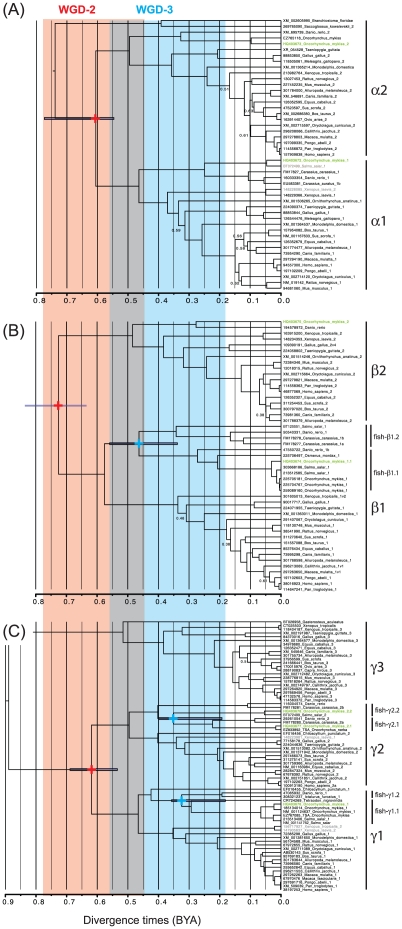
Joint-dated phylogeny of the AMPK subunits. (A) subunit α; (B) subunit β; (C) subunit γ. Sequences obtained in this study are in bold and green; mis-annotated database sequences (NCBI) are in gray. Posterior probabilities are indicated only when <0.70 (all the others are ≥0.70). Error bars (95% Highest Posterior Densities or HPDs) are given only for two genome-wide duplication (GWD) events when these are still visible by the pattern of paralogs: the second round (GWD-2) in vertebrates and the fish-specific event (GWD-3). Mean posterior estimates are indicated by “+” signs. The 95% HPDs of these GWD events are shaded in red and blue, respectively and were constructed from the union of HPDs taken over all detected paralogs across all three subunits. TSA: Transcriptome Shotgun Assembly.

The relaxed clock analysis (**Fig. 1**) reconstructed a rooted tree that clearly shows the whole-genome duplication (WGD) events from which emerged the paralogs of the three subunits. Quite strikingly, most paralogs emerged at the occasion of the same WGD event, here dated between ∼0.775 and 0.450 BYA (Mid-Cryogenina to Upper Ordovician), which corresponds to the second round of WGD that took place in the vertebrates. Subunit-specific singularities exist though. The α subunit, which is the only one for which we found non-vertebrate chordates in GenBank, shows that the duplication giving rise to the α1 and α2 subunits took place after the Chordata/Hemichordata split (**Fig. 1A**). As a result, it might be misleading to classify the *Branchiostoma sp.* and *Saccoglossus sp.* sequences as “α2” (as they are) or even “α1”, as they are neither. The dearth of fish sequences for the α subunit makes it difficult to interpret the phylogenetic dynamics of this subunit here. On the other hand, the β subunit clearly shows evidence for the fish-specific WGD event, here dated between ∼0.560 and 0.180 BYA, as all the gene duplicates seem to have been preserved (Ediacaran to Lower Carboniferous; **Fig. 1B**). Note that the β1.1 sequence obtained in this study (our nomenclature) is of poor quality, which explains its odd placement on the tree (with a very high probability); yet, this does not affect the results of this phylogenetic analysis thanks to the wide sequence sampling adopted here. These two fish-specific β subunits highlight the difficulty noted in the case of α (and of γ below) regarding the interpretation of the dynamics of these genes in fish where subsequent genome duplication events and differential gene loss can affect the shape of the “fish gene” clades. For instance, tentative evidence for the salmonid-specific WGD-4 can be seen in the fish α1 (between HQ403672 and BT072499), γ1.2 (between HQ403676 and NM_001141762) and γ2.2 (between HQ403678 and BT072499) divergences that occurred around 0.100 to 0.025 BYA, which is consistent with previous estimates [28]. Finally, the history of the γ subunit appears to have much deeper roots than that of the two other subunits (**Fig. 1C**). The (γ1,γ2)/γ3 split is very ancient, probably dating back to the first vertebrate WGD event (around 1 BYA). The γ1/γ2 divergence occurred at the same time the paralogs of the α and β subunits emerged, during the second WGD event. In spite of being highly supported, the phylogeny of the fish-specific genes does not reflect the species tree. In particular, the phylogenetic reconstruction seems to be positively misleading for the fish-specific γ2 paralogs, whose identification in **Figure 1C** should therefore be taken as an approximation. The general phylogenetic pattern can however be explained by differential gene loss after the fish-specific WGD event. Nonetheless, α1/α2, β1/β2 and γ1/γ2 all diverged at the same time, at the occasion of the vertebrate second WGD event.

### AMPK phosphorylation status

**Figure 2:**
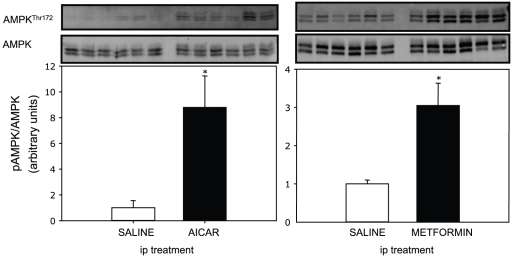
Effect of intraperitoneal (ip) administration of saline solution alone or containing AICAR (1.2 g·kg^−1^) or metformin (120 mg·kg^−1^) on rainbow trout liver AMPK phosphorylation status (at threonine 172; Western blot analysis). Fish were fasted (48 h) prior to injection and livers were sampled 2 h after injection. Gels were loaded with 20 µg total protein per lane. Protein (total AMPK) and phosphorylation levels (AMPK Thr^172^) were normalized to tissue β-tubulin levels. Results are expressed as means ± s.e.m. (*n* = 6) and were analyzed by one-way ANOVA followed by Student-Newman-Keuls comparison test. *Significant difference (*α*<0.05).

The phosphorylation status of AMPK (threonine-172) and the total AMPK levels in liver of fasted rainbow trout after ip injection of AICAR or metformin is reported on **Figure 2**. Although in both experiments the AMPK Thr^172^ phosphorylation status was higher after treatment, the effect exerted by metformin (3-fold increase) was lower than that of AICAR (up to 10-fold increase). No changes were found in the total level of AMPK.

**Figure 3:**
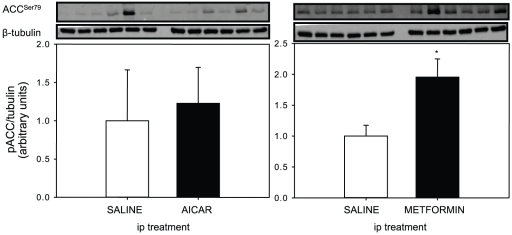
Effect of intraperitoneal (ip) administration of saline solution alone or containing AICAR (1.2 g·kg^−1^) or metformin (120 mg·kg^−1^) on rainbow trout liver ACC phosphorylation status (at serine 79; Western blot analysis). Fish were fasted (48 h) prior to injection and livers were sampled 2 h after injection. Gels were loaded with 20 µg total protein per lane. Protein and phosphorylation levels were normalized to tissue β-tubulin levels. Results are expressed as means ± s.e.m. (*n* = 6) and were analyzed by one-way ANOVA followed by Student-Newman-Keuls comparison test. *Significant difference (*α*<0.05).

The phosphorylation status of ACC (serine-79) in liver of fasted rainbow trout after ip-injected AICAR or metformin is reported on **Figure 3**. AICAR injection had no effect on the phosphorylation status of ACC Ser^79^, although we did observe significant variability between fish for this analysis. In contrast, metformin injection resulted in a significant two-fold up-regulation of the ACC phosphorylation status compared to the saline control group.

**Figure 4:**
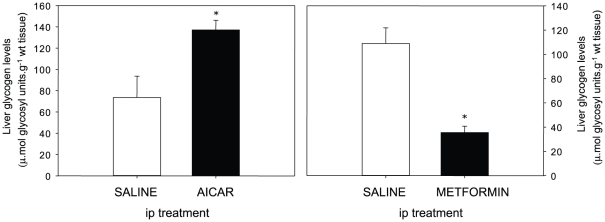
Effect of intraperitoneal (ip) administration of saline solution alone or containing AICAR (1.2 g·kg^−1^) or metformin (120 mg·kg^−1^) on rainbow trout liver glycogen levels. Fish were fasted (48 h) prior to injection and livers were sampled 2 h after injection. Results are expressed as means ± s.e.m. (*n* = 6) and were analyzed by one-way ANOVA followed by Student-Newman-Keuls comparison test. *Significant difference (*α*<0.05).

Hepatic glycogen levels in trout ip-injected with either AICAR or metformin are shown in **Figure 4**. When compared with the saline control group, fish receiving AICAR showed significantly higher glycogen levels. In contrast, a significant decrease (up to 4-fold) in glycogen levels occurred in fish receiving the metformin injection.

**Figure 5:**
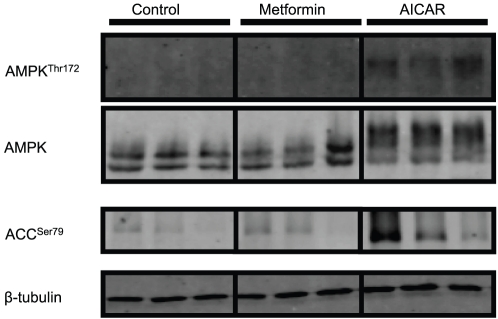
AMPK and ACC phosphorylation status, AMPK, ACC, β-tubulin total protein levels on rainbow trout hepatocyte cell culture 16 h after stimulation with AICAR (0.5 mM) or metformin (0.5 mM). Gels were loaded with 20 µg total protein per lane. Western blots were performed on three individual samples and similar results were obtained.

Analysis of the phosphorylation status of AMPK and ACC as well as total AMPK and β-tubulin levels in cultured hepatocytes of rainbow trout is shown in **Figure 5**. AMPK phosphorylation was only detected in hepatocytes stimulated with AICAR, while no detectable signal was found in either saline control or metformin-treated cells. However, total AMPK protein was found in all treatments, although the bands observed in the group stimulated with AICAR were shifted, possibly as a result of changes in the molecule when phosphorylated. Similar to the results found for the phosphorylated AMPK, the phosphorylation status of ACC (detected in all cells) increased only when AICAR was added to the culture medium. No changes were found in the housekeeping protein β-tubulin among treatments.

**Figure 6:**
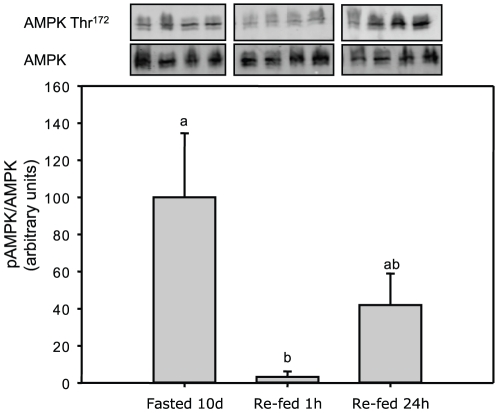
AMPK phosphorylation status and total AMPK protein levels in rainbow trout fasted for 10 days and re-fed once and assessed 1 and 24 h after the meal. Gels were loaded with 20 µg total protein per lane.

The phosphorylation status of AMPK at Thr^172^ and total AMPK levels in trout fasted for 10 days and re-fed is shown in **Figure 6**. The levels of the phosphorylated AMPK were strongly down-regulated in re-fed fish sampled 1 h after the meal (∼50-fold), while at 24 h the phosphorylation status of the kinase in the re-fed fish were intermediate between the fasted and re-fed 1 h groups.

**Figure 7:**
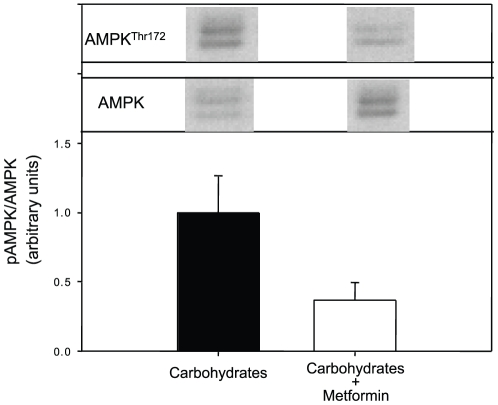
AMPK phosphorylation status (at threonine 172; Western blot analysis), mRNA levels of AMPKα1 and AMPKα2 and glycogen levels in livers of rainbow trout fed with 30% carbohydrates or 30% carbohydrates + metformin (0.25%) for 3 days. Gels were loaded with 20 µg total protein per lane. Protein (total AMPK) and phosphorylation levels (AMPK Thr^172^) were normalized to tissue β-tubulin levels. mRNA levels were estimated using real-time RT-PCR. Expression levels were normalized to β-actin-expressed transcripts which did not change under the experimental conditions and are presented as fold-change against the saline solution-treated group set to 1. Results are expressed as means ± s.e.m. (*n* = 6) and were analyzed by one-way ANOVA followed by Student-Newman-Keuls comparison test. *Significant difference (*α*<0.05).

The phosphorylation status of AMPK at Thr^172^ and the total AMPK protein levels, mRNA levels of AMPK α1 and α2 as well as glycogen levels in the liver of rainbow trout fed with a high carbohydrate diet with or without metformin are shown in **Figure 7**. While no significant differences were noted for glycogen levels, AMPK phosphorylation status was significantly depressed by metformin feeding (mostly due to the increased in the total AMPK content) while mRNA levels of AMPKα1 were significantly up-regulated in these same fish.

### Enzyme transcript abundance

mRNA transcript levels for proteins involved in glucose and lipid metabolism after AICAR or metformin treatments (*in vivo* and *in vitro*) are shown in **Table 2**. GK mRNA expression level was significantly down-regulated both *in vivo* and *in vitro* in the presence of AICAR, which probably was also responsible for AMPK phosphorylation. However, when metformin was administrated, GK mRNA expression level changed only in the *in vivo* experiments, possibly as a result of the differences between the hepatocytes that were cultured in media containing insulin and glucose, while the fish used in the *in vivo* trial were fasted for 48 h. Similar to the changes found for GK, G6Pase mRNA transcript abundance was also reduced when compared with the control in both AICAR trials. In contrast, no changes were observed with the metformin treatment, either *in vivo* or *in vitro*. Two enzymes involved in gluconeogenesis, FBPase and PEPCK, demonstrated no changes for any treatment or for either *in vivo* or *in vitro* experiments. A contrary effect of the AICAR treatment was found comparing the data obtained *in vivo* and *in vitro* regarding the FAS mRNA levels. Thus, FAS mRNA expression levels were significantly up-regulated *in vivo*, but down-regulated *in vitro*. No significant differences in FAS mRNA abundance were found with the metformin treatment, although a general trend to down-regulate was observed.

**Table 2:** *In vivo* (2 h after ip administration) and *in vitro* (16 h of incubation) effects of AICAR (1.2 g·kg^−1^ or 0.5 mM in the medium) or metformin (120 mg·kg^−1^ or 0.5 mM) in rainbow trout on mRNA transcript levels of hepatic genes representative of glucose and lipid metabolism.

	*In vivo*			*In vitro*		
	Control	AICAR	Metformin	Control	AICAR	Metformin
GK	1.05±0.2	0.32±0.06*	18.48±2.03*	1.06±0.2	0.32±0.05*	0.89±0.12
G6Pase	1.12±0.11	0.51±0.05*	1.02±0.09	1.52±0.71	0.89±0.38*	1.38±0.49
FBPase	1.10±0.18	1.50±0.19	1.43±0.21	1.03±0.16	0.90±0.12	1.08±0.13
PEPCK	1.16±0.19	1.17±0.23	0.53±0.11	1.03±0.15	1.02±0.18	1.00±0.20
FAS	1.09±0.16	2.51±0.21*	0.67±0.17	1.05±0.19	0.29±0.05*	096±0.16

GK (glucokinase), G6Pase (glucose 6-phosphatase), FBPase (fructose 1,6-bisphosphatase), PEPCK (phospho*enol*pyruvate carboxykinase), FAS (fatty acid synthase) Results are expressed as means ± s.e.m. (n = 6) and were analyzed by one-way ANOVA followed by Student–Newman–Keuls comparison test.

*Significant difference (*P*<0.05). mRNA levels were estimated using real-time RT-PCR and normalized to elongation factor 1α (EF1α) transcripts which did not change under the experimental conditions.

Data are presented as relative ratios of the target gene to the reference gene (EF1α).

## Discussion

### Phylogenetic analysis

Our phylogenetic analysis clearly shows the near-simultaneous emergence of the AMPK subunit paralogs at the occasion of the second vertebrate-specific WGD event. The fish-specific WGD event led to the emergence of additional paralogs, retained for the β1, γ1 and γ2 subunits. We could not find evidence for any other fish-specific paralogs, be it in GenBank or through our sequencing effort in rainbow trout, which does not mean however that these sequences have been lost. Note that no fish-specific paralogs were found in the α subunit, which might be linked to the presence of the activation site at Thr^172^ and to the conserved function of AMPK across vertebrates (see below for experimental evidence). Although we present here the first attempt at a phylogenetic analysis of this important nutrient sensor, deeper sequencing efforts are required to unravel the complex history of AMPK subunits in fish models such as the rainbow trout, and its relationship with the emergence of AMPK functions.

### AICAR and metformin-induced AMPK phosphorylation in trout liver and hepatocytes

Our study demonstrates for the first time in rainbow trout the presence of AMPK in its total form and, more importantly, the phosphorylation at the Thr^172^ of the α subunit. The binding of AMP to the γ subunit activates AMPK *via* a complex mechanism involving direct allosteric activation and phosphorylation of the α subunit on the Thr^172^ by upstream kinases such as the protein kinase LKB1 and the CaMKKIIβ [1]. Our first objective was to activate AMPK in trout liver to evaluate the metabolic events triggered by activation of this kinase. To do so, we developed two *in vivo* experiments, injecting fasted trout with two mammalian activators of AMPK, AICAR [29] and metformin [11]. Both AICAR and metformin were able to induce AMPK phosphorylation in trout liver at 2 h post-drug administration. This is the first evidence that phosphorylation of AMPK can be pharmacologically-induced in a fish. Two bands of the expected molecular weight (∼65-70 kDa) were detected, consistent with the two AMPK α-isoforms present in rainbow trout in which a threonine at position 172 (Thr^172^) was found (see GenBank accession HQ403672 and 403673). The fact that these same drugs are able to activate AMPK in other animals including birds [30], [31] and mammals [29] supports the conserved nature of AMPK function, at least in the vertebrates. The activation of AMPK by metformin traditionally used as an anti-diabetic drug in the trout model constitutes a novel finding [32]. Musi et al. [33] did demonstrate that metformin stimulated AMPK activities in skeletal muscle of rats with type 2 diabetes. The activation of AMPK by metformin has been elusive in fish, as after the administration of this drug in rainbow trout (infused ip at relatively low doses, 20 mg·kg^−1^·day^−1^) the phosphorylation of this kinase was not detected [13]. Unfortunately, in other studies in which metformin was administrated to fish, including zebrafish (transdermally [34]) or common carp (ip [12]), AMPK activation was not explored. Additionally, we present in this study the phosphorylation status of AMPK in fish fed a high carbohydrate diet supplemented with metformin. After 3 days of feeding, no activation of AMPK was found even though mRNA expression levels of the α1 subunit (the major α isoform in liver) and the total AMPK expression levels (data not shown) were up-regulated. The fact that in similar feeding studies with trout (e.g., [14]), metformin did result in normoglycemia in high-carbohydrate fed fish and the lack of AMPK activation in the present study, reinforces the idea that the normoglycemic effect of metformin in trout is AMPK-independent. The fact that the phosphorylation of AMPK was higher after AICAR administration than with metformin may relate to the dose employed being 10-fold higher for AICAR. However, the mechanism through which each drug activates AMPK is in fact different. AICAR is known to be phosphorylated to 5-amino-4-imidazolecarboxamide ribotide (ZMP), which is an analogue of AMP and mimics several of the cellular effects of adenine, including replacing 5′-AMP as an AMPK activator [35]. Metformin, however, stimulates LKB1 kinase, which in turn, phosphorylates AMPK [11]. LKB1 phosphorylation status at Ser^428^ was evaluated in the present study, but no bands for LKB1 were found in trout liver or hepatocytes homogenates (data not shown).

Further support for the induction of AMPK phosphorylation in trout liver was found using hepatocytes primary cultures stimulated with either AICAR or metformin. Consistent with the *in vivo* study, AICAR after a 16 h incubation induced AMPK phosphorylation. However, no AMPK phosphorylation was found in the metformin-stimulated hepatocytes in clear contrast with the ip *in vivo* results. Since the antibody used in the present study is not specific for trout, it is possible that AMPK phosphorylation was below the detection level of the particular antibody used. However, we can also hypothesize that the dose used for the stimulation was too low since studies with mammalian hepatocytes show maximum AMPK phosphorylation was achieved at 2 mM metformin [26], or 4 times-higher than used in the present study.

Finally, we assessed the phosphorylation status of liver AMPK in trout subjected to a fasting-re-feeding trial. After 10 days of food-deprivation trout were re-fed and 1 h later, the AMPK phosphorylation status was significantly decreased, while 24 h after this first meal the levels trend to recover to those of the fasted fish. Although we are unaware whether a 10-day fast is adequate to cause energy stress in this species which is able to fast for weeks [36], the down-regulation of AMPK phosphorylation after the first meal matches the expected inhibition of this kinase under energy surplus conditions demonstrated in mammals [1]. The fact that AMPK can be dynamically (de-)phosphorylated in trout liver under physiological conditions supports the idea that this kinase exerts a relevant role on fish metabolism in accordance with its previously reported function in hypoxic cyprinids [4], [5], [6]. Whether AMPK is working as an energy sensor key to rainbow trout liver function or not requires further studies.

### Indirect evidence of AMPK activity in trout liver and hepatocytes

One of the main downstream targets of mammalian AMPK is the lipogenic enzyme ACC. ACC-serine79 is directly phosphorylated by this kinase to inhibit its activity [37]. Consequently the phosphorylation status of ACC is also considered as an indirect indicator of AMPK activity [38]. Only metformin in our *in vivo* trials induced a significant increase in phospho-ACC protein levels while a slight increase was noted in phospho-ACC in trout hepatocytes, suggesting some degree of AMPK activation, possibly not detected in our present conditions as discussed above. The *in vivo* effect of metformin is not only consistent with the increased phosphorylation status of the AMPK, but also with the ability of this drug to repress lipogenesis in the liver of mammalian species [39] and our recent studies in trout. Our study showed that long-term treatment of trout with metformin did not stimulate AMPK phosphorylation in the liver, but it did up-regulate the lipogenic potential, including FAS gene expression and activity [13], [14]. These data confirm the ability of AMPK to exert a similar control on lipogenesis in fish as in mammals [39], but also that the increased lipogenesis observed after chronic metformin treatments in trout is probably AMPK-independent [13], [14]. Surprisingly, no *in vivo* induction of ACC phosphorylation was found in the present study, although as noted the AICAR trial showed considerable variability. However, the results obtained in AICAR-stimulated hepatocytes strongly support an increased ACC phosphorylation that resulted from an AICAR-dependent AMPK activation. As stated above, the ACC phosphorylation is consistent with a decreased ACC activity and also with the down-regulated mRNA levels of FAS observed in our previous *in vivo* studies [40] and as described in mammals [41]. The fact that FAS gene expression is increased *in vivo* after AICAR injection is surprising and could be also related with the pharmacological AICAR dose used in the present study. Other metabolic markers addressed here have not previously been explored in fish before with AMPK activation, and constitute important data concerning potential metabolic AMPK functions in fish.

### Metabolic events related with the AMPK phosphorylation in rainbow trout

AICAR effects on metabolic-related genes in liver and hepatocytes of trout other than FAS discussed above can be summarized as the down-regulation of GK and G6Pase mRNA expression levels, while the gluconeogenic genes assessed (PEPCK and FBPase) were unaffected. The results obtained both *in vivo* and *in vitro* are internally consistent, re-enforcing the idea that the AICAR-induced AMPK activation is the main mediator of such a regulation. Moreover, our results are in agreement with the main function of AMPK in mammals where it acts as a master regulator of energy production, repressing anabolic and stimulating catabolic processes [1]. Thus, the down-regulation of G6Pase observed here is consistent with the AICAR induced-inhibition of gene expression of this enzyme described in mammals [42]. Similarly, the repression of GK, considered as one of the key regulators of both glycogenesis and lipogenesis in liver [43], is consistent with the above-mentioned roles of AMPK in mammals.

Although these data are consistent with the results obtained for glycogen levels in AICAR-injected trout, they are in clear disagreement with those reported in mammals where AMPK α activation leads to the inhibition of glycogen synthase [44]. However, in mice [45], as in the present study (data not shown), acute AICAR administration promoted significant hypoglycemia, probably triggering the counter-regulatory response that resulted in the depletion of the hepatic glycogen stores [46]. Concerning glycogen levels in metformin-treated trout, the regulation appears more complex. When administrated ip in trout, we found that metformin decreased glycogen levels, as reported in mammals [47] and consistently with AMPK activation [44]. However, no hypoglycemic effects of the drug were found in our study, in contrast with reduction in plasma glucose levels reported 1 h after ip-metformin administration in rats [48]. On the other hand, chronic metformin treatment either did not alter AMPK activation [40] or reduced it (present study), even though hypoglycemia is systematically observed. Consistently with these results, we suggest that the hypoglycemic effect of metformin in trout is not linked to AMPK activation, while some of the effects directly linked to the metformin-dependent AMPK activation (i.e. changes in glycogen) were not observed when hypoglycemia appeared and AMPK was not activated.

### Conclusions and Perspectives

We clearly demonstrate in this paper the presence of AMPK in rainbow trout. In particular we present evidence regarding the retention of paralogs that emerged during the fish-specific whole-genome duplication event for the β and γ subunits, while the α subunit does not seem to show any evidence of such a duplication event, presumably because of the loss of the corresponding paralog. This loss might be in relation to the functional role of the α subunit, a possibility that should be investigated by extended sequencing studies. We also show for the first time evidence for AMPK activation through the phosphorylation of the AMPK α at Thr^172^, and changes in phosphorylation pattern under physiological and pharmacological conditions. Moreover, the phosphorylation of this kinase by two recognized AMPK activators (AICAR and metformin) does induce changes in the main downstream target, the phosphorylation of ACC, as well as changes in glycogen levels and in several molecular actors involved in hepatic glucose and lipid metabolism. Further confirmation of our results can be found in the effects exerted mainly by AICAR in trout hepatocytes and the decreased AMPK phosphorylation in re-fed trout. The fact that many of our *in vivo* and *in vitro* results are consistent with the recognized role of AMPK as a master regulator of energy homeostasis in organisms [1], [2] supports the idea that these functions are conserved also in the piscine model. Further studies, however, must be conducted in order to clarify the AMPK-related functions on fish metabolism. As a link between glucagon and lipids in the regulation of hepatic AMPK signaling was recently found [49], further studies are needed to link hormone levels with this key signaling process.
